# Supplementary material for: Knowledge, Attitudes, and Practices of Iraqi Parents Regarding Antibiotic Use in Children and the Implications
Source: Antibiotics (Basel). 2025 Apr 3;14(4):376. doi: 10.3390/antibiotics14040376 (PMC12024425; doi:10.3390/antibiotics14040376)
Supplement: Supplementary file 1 [file antibiotics-14-00376-s001.zip › antibiotics-3551128-supplementary.pdf]

Table S1 – KAP Questionnaire

Knowledge, Attitudes, and Practices of Iraqi Parents Regarding Antibiotic Use in Children and the implications

If you agree to be interviewed by the researcher, you consent to participate in this study.

You will not be asked for any information that reveals your identity.

Q1. Demographic characteristics of the study sample

Age of parent ☐ 19–29 ☐ 30–39 ☐ 40–49 ☐ ≥50

Gender ☐ Male ☐ Female

Place of living; City --------------- Remote area -------------------

Education level of the father; ☐ High school or lower ☐ University degree or higher

Education level of the mother ☐ High school or lower ☐ University degree or higher

The family income per month

☐ Less than 500,000 ID (Low)

☐ 500,000–1,000 ,0000 ID (Moderate)

☐ 1,000 ,0000—1,500,000 ID (High)

Does the father or mother work in the medical field? ☐ Yes ☐ No

Number of children ☐ 1 ☐ 2–3 ☐ ≥4

Does one of the children complain of a chronic disease (URTI, UTI, or others) that requires frequent use of antibiotics? ☐ Yes ☐ No

Chronic disease ……………………

Q2. Parental knowledge, attitudes, and practices

Q2.1. Parental knowledge on antibiotics indications and side effects (Agree, do not know, Disagree)

☐ Most symptoms of cough, influenza and cold that affect children result from viral infection.

☐ Antibiotics are used to treat bacterial infections.

☐ Antibiotic use may cause side effects such as diarrhoea or allergy.

☐ Giving antibiotics to the child without a prescription may expose him to side effects.

Q2.2-Parental attitudes towards antibiotics use (Agree, do not know, Disagree)

- The physician is the only healthcare provider who should prescribe antibiotics
- The antibiotic which I used after the physician's prescription will be always effective to treat similar symptoms
- I prefer to give an antibiotic to my children rather than wait until he becomes better without it
- Antibiotics are used until symptoms resolve
- I keep antibiotics or leftover antibiotics at home for emergency conditions.
- When a physician does not prescribe antibiotics for symptoms of a common cold, nasal congestion or flu for the child, you are pleased with his prescription

Q2.3. Antibiotics self-prescription practice among study participants

Did you give antibiotics to your child without a prescription previously? ☐ Yes ☐ No

At what age?

☐ <1 year ☐ 1–5 years ☐ 6–10 years ☐ 11–12 years

Q.2.4 Parents gave antibiotic to:

☐ Treat the disease ☐ Prevent disease occurrence ☐ Both answers

Q.2.5 The main factor to choose a specific type of antibiotic was

☐ Require less number of times daily
☐ Require less number of days
☐ Acceptable taste
☐ Cost

Q2.6 If your child feeling well, did you stop your child medication before the end of the antibiotic prescribed course ☐ Yes ☐ No

Q.2.7 Did you give you child an extra dose (more than the prescribed doses by the physician)?

☐ Yes ☐ No

Q.2.8 Did you give you child more than one antibiotic in one time without medical advice (injection plus suspension)? ☐ Yes ☐ No

Reasons ……………………………………………………

Q.3 Sources of antibiotics used

☐ Purchased from pharmacy with prescription
☐ Purchased from pharmacy without prescription
☐ Purchased from other places

Q.4 Reasons for not consulting the physicians;

☐ Symptoms are not severe and do not require physician consultation
☐ I have a previous experience with drug efficacy
☐ Lack of time ☐ Lack of money ☐ Others

Q.5 You give your children antibiotics

☐ Always ☐ Frequently ☐ Sometimes ☐ Never

Q.6 Medical conditions for which antibiotics were used

☐ Fever ☐ Diarrhoea ☐ Cough and the common cold ☐ Ear pain
☐ Other conditions

Q. 7 Source of information for antibiotic use

☐ Pharmacist ☐ Nurse ☐ Medication packages ☐ Friends
☐ Social media ☐ Previous experience
